# Proposing a validated clinical app predicting hospitalization cost for extracranial-intracranial bypass surgery

**DOI:** 10.1371/journal.pone.0186758

**Published:** 2017-10-27

**Authors:** Hai Sun, Piyush Kalakoti, Kanika Sharma, Jai Deep Thakur, Rimal H. Dossani, Devi Prasad Patra, Kevin Phan, Hesam Akbarian-Tefaghi, Frank Farokhi, Christina Notarianni, Bharat Guthikonda, Anil Nanda

**Affiliations:** 1 Neurosurgery, Louisiana State University Health Sciences Center, Shreveport, Los Angeles, United States of America; 2 NeuroSpine Surgery Research Group (NSURG), Barker St Randwick, Prince of Wales Private Hospital, Sydney, Australia; University of Pennsylvania Perelman School of Medicine, UNITED STATES

## Abstract

**Object:**

United States healthcare reforms are focused on curtailing rising expenditures. In neurosurgical domain, limited or no data exists identifying potential modifiable targets associated with high-hospitalization cost for cerebrovascular procedures such as extracranial-intracranial (ECIC) bypass. Our study objective was to develop a predictive model of initial cost for patients undergoing bypass surgery.

**Methods:**

In an observational cohort study, we analyzed patients registered in the Nationwide Inpatient Sample (2002–2011) that underwent ECIC bypass. Split-sample 1:1 randomization of the study cohort was performed. Hospital cost data was modelled using ordinary least square to identity potential drivers impacting initial hospitalization cost. Subsequently, a validated clinical app for estimated hospitalization cost is proposed (https://www.neurosurgerycost.com/calc/ec-ic-by-pass).

**Results:**

Overall, 1533 patients [mean age: 45.18 ± 19.51 years; 58% female] underwent ECIC bypass for moyamoya disease [45.1%], cerebro-occlusive disease (COD) [23% without infarction; 12% with infarction], unruptured [12%] and ruptured [4%] aneurysms. Median hospitalization cost was $37,525 (IQR: $16,225-$58,825). Common drivers impacting cost include Asian race, private payer, elective admission, hyponatremia, neurological and respiratory complications, acute renal failure, bypass for moyamoya disease, COD without infarction, medium and high volume centers, hospitals located in Midwest, Northeast, and West region, total number of diagnosis and procedures, days to bypass and post-procedural LOS. Our model was validated in an independent cohort and using 1000-bootstrapped replacement samples.

**Conclusions:**

Identified drivers of hospital cost after ECIC bypass could potentially be used as an adjunct for creation of data driven policies, impact reimbursement criteria, aid in-hospital auditing, and in the cost containment debate.

## Introduction

United States healthcare expenditure as a fraction of its Gross Domestic Product (GDP) is the highest compared to any other nation, translating to over 17 percent of its GDP in the recent years. [1, 2] In 2015 alone, official estimates suggest healthcare spending exceeded $3.2 trillion, creating a net increase of 5.8% from the preceding year, and over 23% since 2010. [2] As witnessed in previous decades, the increment in healthcare spending has consistently outpaced the annual GDP growth. In this context, several initiatives directed towards cost containment are implemented to redefine value in healthcare.[3] Pertinent developments include replacement of the “fee-for-service” model with bundled payments, administrative restructuring for improved efficiency, Medicaid expansion, monitoring by accountable care organizations (ACOs), and imposing financial penalties on hospitals and providers for inadequate care as determined by readmission rates. Concerning these seismic political reforms in healthcare setup, neurosurgical procedures particularly those involving cerebral vasculature are likely to elicit attention from policy makers owing to the high risks and hospitalization costs associated with it.

Extracranial-intracranial (EC-IC) bypass is a commonly indicated cerebral revascularization technique in treating moyamoya angiopathy and complex aneurysmal repair especially those with a broad neck, narrow lumen with perforating arteries, amenable to surgical clipping. Other indications, albeit rare, include resection of skull base tumors encasing the ICA, and small cohort of patients with cerebro-occlusive disease (COD) that are refractory to medical management, although its utility in the latter has been fairly limited following results from the ECIC bypass trial.[4] Critics believe outcomes would have been different with apropos patient selection and randomization method, and well-defined control arm.[5–8] Despite its limited indication, cerebrovascular surgeons deem it as a valuable armamentarium that is often a necessary alternative for a select cohort of patients. Amin-Hanjani et al demonstrated a 400% increase in relative caseload for bypass between 1992 and 2001.[9] Further, it is argued that bypass procedures might be resurrected for patients with major artery stenosis or occlusion or both with decompensated hemodynamic failure, after recent studies demonstrate remarkable benefit.[10] Proponents of bypass surgery demonstrate satisfactory outcomes in treating select patients with giant intracranial and complex aneurysms.[10–13] Some focused on improvising the technique, redefining patient selection, or evaluating its safety and efficacy in the modern endovascular era,[12, 14] while others focused on epidemiological trends using administrative databases.[9, 15–17] Efforts by NeuroPoint Alliance are underway to formulate a prospective registry to assess outcomes following ECIC bypass for carotid occlusion. However, limited or no literature exists on drivers of hospitalization costs for patients undergoing bypass procedure.

To this effect, the present study focused on investigating potential drivers of initial hospitalization cost for patients undergoing ECIC bypass. Baseline identification of modifiable factors associated with costs would provide deeper economic insights about the procedure and care, with the hope of aligning practices and adopting measures to make it economically viable. Additionally, we propose a validated clinical apparatus predictive of hospitalization cost following ECIC bypass.

## Methods

### Data source

We utilized data obtained from the Healthcare Research and Quality (HCUP) Nationwide Inpatient Sample (NIS) database for the years 2002 through 2011. The NIS is developed by the Agency for Healthcare Research and Quality (AHRQ, Rockville, Maryland).[18] It is the publicly available largest inpatient cohort that includes all-payer. When unweighted, it contains 5 to 8 million discharge-level records each year from over 1000 participating non-federal hospitals from several states, representative of stratified, random sampling depicting 20% discharges across hospitals.[18] The clinical data for the years studied is encoded using the International Classification of Diseases, 9th Revision, Clinical Modification (ICD-9-CM) codes into several diagnoses and procedures.[19] A detailed overview of the database can be accessed at http://www.hcup-us.ahrq.gov/nisoverview.jsp.

### Cohort definition

**Fig 1:**
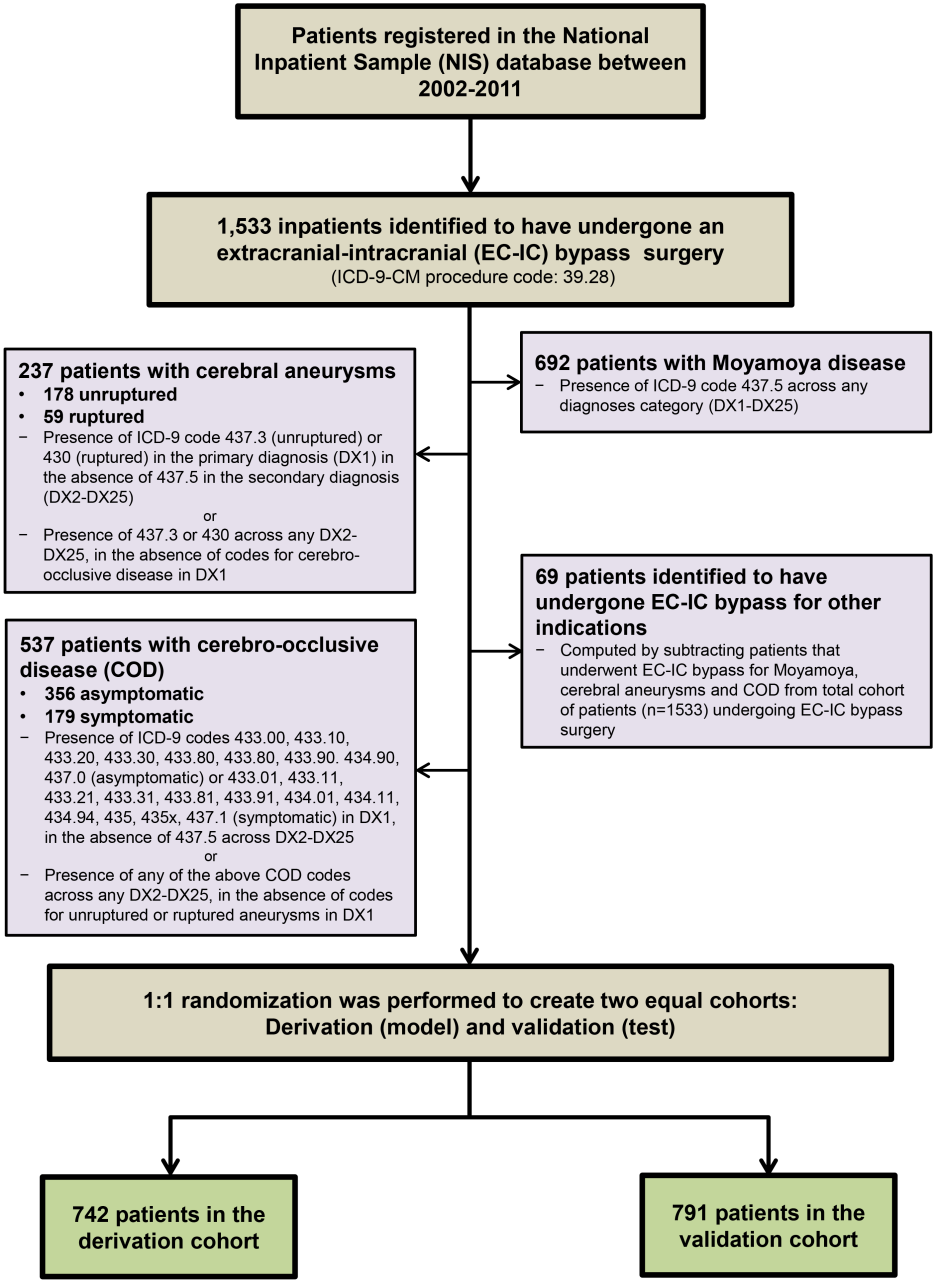
Algorithm for cohort selection for patients undergoing EC-IC bypass.

Records of patients registered in the database that underwent an EC-IC bypass surgery during the study years were identified using the presence of ICD-9-CM procedure code 39.28 (Fig 1). Common indications for bypass surgery were identified as depicted in the selection algorithm using appropriate coding definitions (S1 Table).

### Outcome measure (endpoint)

The primary outcome measure was total hospitalization cost following EC-IC bypass surgery. To obtain cost data, hospital charges were converted using the HCUP group average cost-to-charge ratios modelled on hospital accounting reports from the Centers for Medicare and Medicaid Services (CMS).[20] The cost data provides estimates on the actual incurred expenses in the production of hospital services including wages, supplies, and utility costs while hospital charges reflect the amount billed by the hospital for the duration of inpatient hospitalization, excluding the physician fees. For each study year, costs were inflation adjusted to the 2015 US dollar value using the national consumer price index.

### Explanatory variables

Pertinent exposures utilized for model development included patient demographics and clinical characteristics, and hospital data. Variable selection primarily relied upon considering their plausible impact on hospitalization cost, centric to their availability in the data source and also based upon the authors experience.

Demographic data included age, sex, race, primary payer, median household income quartiles for area ZIP code of residence and admission status (elective vs non-elective). Clinical characteristics included indication for bypass surgery along with 14 comorbidities and 8 adverse events likely to impact costs (S1 Table). The latter included procedure related complications such as postoperative neurologic complications including stroke, wound infections, postoperative cardiac or respiratory complications, hydrocephalus, and general post-operative complications impacting hospitalization stay such as deep venous thrombosis (DVT), pulmonary embolism, wound complications, and acute renal failure. Hospital data included hospital-level characteristics such as bed size, academic status, region, hospital volume, and hospital stay specific factors potentially known to impact costs such as total number of diagnoses record (NDX) and procedures (NPR), days to bypass surgery, and post-bypass length of stay (LOS).

For computing hospital volume, unique hospital identifier numbers for individual case records were used. Weighted estimates were obtained to identify number of bypass surgeries across centers (total hospitals = 218) over the 10-year study period. Based on the number of bypass procedures performed per hospital, centers were labelled as a low-volume center (LVC) if it performed 10 or less (1/year on average), or a medium volume center (MVC) if 11 to 200 procedures (2-20/year), or a high-volume center (HVC) if more than 200 bypass (>20/year) over the 10-year study period. Subsequently, each case was labelled as having undergone a bypass at a LVC (108 hospitals), or an MVC (104 hospitals) or a HVC (6 hospitals). These volume assignments and cutoff values were selected preferentially selected based upon the skewed distribution of volumes across centers performing bypass (S1 Fig).

### Statistical analysis

Model development and internal validation relied primarily upon a split sample approach. For this purpose, a 1:1 randomization of the study cohort was performed to generate 2 independent cohorts, the derivation (test) and the validation (training). Differences in categorical variables across these 2 cohorts were assessed using the Pearson’s χ^2^ test or Fisher’s exact test as appropriate, while differences in metric values were analyzed using independent samples t-test or the non-parametric Mann-Whitney U test. Categorical values are reported as frequencies and proportions, while metric as mean ± SD and/or median (IQR).

Prior to constructing an ordinary least square model to identify drivers of hospital costs, assessment of cost data demonstrated a remarkable degree of non-Gaussian distribution. To mitigate heteroskedastic variance in errors by modelling cost data using OLS, transformation of costs using several methods were attempted, of which the natural logarithm (ln) bestowed best fit. Similarly, NDX and NPR were positively skewed, and ln transformation provided best fit to normality. Further, we noted missing values for explanatory variables in our data (S2 Table). To effectively deal with missing values that could potentially introduce bias in our estimates, a model-based multiple imputation (mi) approach was preferred over alternative approaches such as traditional deletion methods or single imputation technique due to its enhanced performance.[21–25] Imputed datasets (n = 5) were introduced in our regression model (S3 Table). For non-binomial categories, we introduced dummy variables in to the model. No collinearity was observed with variance-inflation factor (VIF) <10. Coefficient of determination (R^2^) was noted. The derived model was internally validated by applying it to the validation cohort, and the R^2^ value was computed. Residual analysis with histograms (S2 Fig: panel A and B), P-P plots (S3 Fig: panel A and B), and scatter plot (S4 Fig) were performed for both the derivation and validation cohort. The predicted estimates for the validation cohort were examined by plotting against the observed values. Goodness of fit was assessed and no heteroskedasticity was noted. For reporting purposes, back-transformation of estimates was performed to assess the plausible impact of the identified drivers of cost as a function of percentage change to the cost value. For clinical utility of our proposed model, a clinical app or calculator to provide an average estimate of hospitalization costs for ECIC bypass procedures is proposed. A link to the online version can be accessed at https://www.neurosurgerycost.com/calc/ec-ic-by-pass.

As a part of sensitivity analysis, we assessed our model using non-imputed datasets and by using bootstrapped estimates using 1000-replacement samples in the development cohort. Estimates from these secondary measures closely resembled our findings from primary analysis, therefore they are not being reported separately. All statistical tests were 2-tailed, and a Type I error set at 5% was considered of statistical significance. All statistical analyses were performed using SPSS version 22.0 (IBM, Armonk, NY) and R Foundation for Statistical Computing (64-bit version R.2.15.3).

## Results

### Patient demographics and characteristics

Between 2002 and 2011, 1,533 patients registered in the NIS underwent an EC-IC bypass procedure across 218 non-federal US hospitals. Overall, the mean age of the cohort was 45.18 ± 19.51 years, and approximately 58% were female (Table 1). Approximately one-third patients were Caucasian (66%; n = 720), and over half were privately insured (53%; n = 814). Most common indications for bypass were observed in patients with moyamoya disease (45%), followed by COD (23% without infarction; 12% with infarction), and unruptured (12%) and ruptured (4%) aneurysms. The average length of hospital stay was 9.77 days, while post-bypass LOS was 7.97 days. Patients undergoing bypass had on an average 8 concomitant diagnoses on record, while the number of inpatient procedure coded was four. Based on our definitions for volume-caseload, we noted that two-thirds of patients (65%; n = 987) underwent the procedure at centers with medium caseload (2-20/year), followed by high-volume centers (27%; n = 418), and the least occurring at LVC (8%; n = 128).

**Table 1:** Demographic and clinical characteristics of 1,533 patients registered in the NIS that underwent EC-IC bypass surgery across 218 non-federal hospitals between 2002 and 2011.

		Overall	Model cohort	Validation cohort	P value
		N	N	N	
**Sample size**		1533	742	791	
**Demographics**					
Age(in years)*	Mean±SD	45.18±19.51	45.48±19.75	44.90±19.30	0.547
*Female sex*,n(%)*		880(57.6)	433(58.7)	447(56.7)	0.425
*Race*,n(%)*	Caucasians	720(65.8)	350(64.6)	370(66.9)	0.416
	African Americans	154(14.1)	82(15.1)	72(13.0)	0.315
	Hispanic	87(7.9)	40(7.4)	47(8.5)	0.494
	Asian	87(7.9)	47(8.7)	40(7.2)	0.379
	Others	47(4.3)	23(4.2)	24(4.3)	0.937
*Primary payer*,n(%)*	Medicare	314(20.5)	160(21.6)	154(19.5)	0.316
	Medicaid	273(17.8)	133(17.9)	140(17.7)	0.917
	Private including HMO	814(53.1)	382(51.5)	432(54.7)	0.21
	Self	42(2.7)	22(3.0)	20(2.5)	0.604
	Others	89(5.8)	45(6.1)	44(5.6)	0.679
*Income*,n(%)*	Lowest quartile	345(23.2)	178(24.7)	167(21.7)	0.183
	Second quartile	379(25.4)	186(25.8)	193(25.1)	0.78
	Third quartile	388(26.0)	178(24.7)	210(27.3)	0.237
	Fourth quartile	378(25.4)	180(24.9)	198(25.8)	0.706
*Elective admission*,n(%)*		1,069(69.8)	521(70.2)	548(69.4)	0.718
**Clinical characteristics**					
***Indications for EC-IC bypass*,*n(%)***	Ruptured aneurysms	59(3.8)	26(3.5)	33(4.2)	0.497
	Unruptured aneurysms	178(11.6)	85(11.5)	93(11.8)	0.854
	COD without stroke	356(23.2)	176(23.7)	180(22.8)	0.655
	COD with stroke	179(11.7)	98(13.2)	81(10.2)	0.071
	Moyamoya disease	692(45.1)	318(42.9)	374(47.3)	0.082
	Others	69(4.5)	39(5.3)	30(3.8)	0.167
***Comorbidities*,*n(%)***	TIA	39(2.5)	20(2.7)	19(2.4)	0.715
	Stroke	267(17.4)	127(17.1)	140(17.7)	0.763
	Seizures	174(11.4)	85(11.5)	89(11.3)	0.9
	Anemia	237(15.5)	114(15.4)	123(15.5)	0.92
	Coagulopathy	39(2.5)	22(3.0)	17(2.1)	0.311
	Hypercholesterolemia	420(27.4)	213(28.7)	207(26.2)	0.266
	Hypertension	802(52.3)	397(53.5)	405(51.2)	0.367
	CAD	225(14.7)	114(15.4)	111(14.0)	0.462
	COPD	192(12.5)	104(14.0)	88(11.1)	0.087
	CRF	27(1.8)	12(1.6)	15(1.9)	0.678
	DM	311(20.3)	159(21.4)	152(19.2)	0.282
	Alcohol abuse	31(2.0)	14(1.9)	17(2.1)	0.715
	Obesity	103(6.7)	47(6.3)	56(7.1)	0.56
	Hyponatremia	86(5.6)	46(6.2)	40(5.1)	0.331
***Complications*,*n(%)***	Neurologic complication	144(9.4)	77(10.4)	67(8.5)	0.201
	Respiratory complication	150(9.8)	73(9.8)	77(9.7)	0.946
	Cardiac complication	54(3.5)	30(4.0)	24(3.0)	0.284
	Hydrocephalus	62(4.0)	30(4.0)	32(4.0)	0.998
	Wound complication	58(3.8)	31(4.2)	27(3.4)	0.433
	Acute renal failure	23(1.5)	≤10	13(1.6)	0.634
	Pulmonary embolism	30(2.0)	13(1.8)	17(2.1)	0.575
	DVT	70(4.6)	32(4.3)	38(4.8)	0.645
**Hospital-level characteristics,n(%)**					
Bed size*	Small	132(8.6)	58(7.8)	74(9.4)	0.287
	Medium	189(12.4)	88(11.9)	101(12.8)	0.596
	Large	1,207(79.0)	593(80.2)	614(77.8)	0.245
Teaching status*	Rural	27(1.8)	17(2.3)	≤10	0.126
	Urban non-teaching	80(5.2)	44(6.0)	36(4.6)	0.222
	Urban teaching	1,421(93.0)	678(91.7)	743(94.2)	0.063
***Region***	Northeast	227(14.8)	111(15.0)	116(14.7)	0.871
	Midwest	402(26.2)	187(25.2)	215(27.2)	0.379
	South	434(28.3)	218(29.4)	216(27.3)	0.368
	West	470(30.7)	226(30.5)	244(30.8)	0.869
***Hospital volume***	LVC	128(8.3)	67(9.0)	61(7.7)	0.351
	MVC	987(64.4)	488(65.8)	499(63.1)	0.273
	HVC	418(27.3)	187(25.2)	231(29.2)	0.079
**Hospital-stay specific factors**					
***LOS(in days)***	Total hospital stay,mean(median)	9.77(5)	9.48(5)	10.03(5)	0.876
	Post-bypass stay,mean(median)	7.97 (4)	7.69(4)	8.24(4)	0.861
***Time to ECIC bypass procedure(in days)***	Mean	1.79	1.79	1.79	0.231
	Median	0	0	0	
***No*. *of diagnoses(NDX)***		7.84(7)	7.92(7)	7.76(7)	0.937
***No*. *of procedures(NPR)***		3.64(2)	3.67(2)	3.62(2)	0.933

*Frequencies and proportions reported after excluding patients with missing values for age (0.1%), gender (0.4%), elective admission (0.1%), race (28.6%), income quartiles (2.8%), payer (0.1%), hospital bedsize (0.3%) and academic status (0.3%) [S2 Table]

Following a 1:1 randomization of the study cohort, characteristically similar subsets were generated that were used for model derivation and validation. Patients in the 2 groups were similar in characteristics, with no significant differences across demographics, clinical and hospital characteristics (Table 1). No differences were observed across the indications for which bypass was performed in the 2 cohorts. The similarity across the 2 groups permitted unbiased testing of our model validity in the independent validation (training) cohort.

### Primary outcome

Overall, the mean hospitalization costs for patients undergoing the bypass procedure was US$ 56,722 (Median: US$37,525) (Table 2). Hospitalization costs were highest for patients undergoing the procedure for cerebral aneurysms as compared to other indications, with ruptured aneurysms noted to have relatively higher costs as compared to patients with unruptured aneurysms (Mean: US$ 168, 311 vs US$ 91,930). For other indications, the average cost for hospitalization following bypass was US$ 75,098 for patients with cerebro-occlusive disease (COD) with infarction, US$ 43,815 for patients with MMD, and the least for patients with COD without stroke (US$ 36,050). No differences in the means overall costs, or costs for above indications were noted in the derivation and validation cohort (Table 2).

**Table 2:** Inflation adjusted hospitalization cost for ECIC bypass surgery*.

Cost data	Overall	Model cohort	Validation cohort	P value
Mean	56722	56228	57188	0.236
95% CI	53501–59942	51632–60825	52665–61711	
Median	37525	35205	39157	
IQR	16225–58825	13398–57012	18044–60271	
**Indications**				
**Ruptured aneurysms**				
Mean	168311	139544	193243	0.07
95% CI	138597–198026	93925–185163	154138–232348	
Median	148352	98202	178181	
IQR	72136–224568	37860–158545	89735–266628	
**Unruptured aneurysms**				
Mean	91930	99185	85084	0.212
95% CI	80806–103055	80913–117456	71835–98333	
Median	67629	71543	65276	
IQR	36522–98736	35752–107334	36679–93873	
**Moyamoya disease**				
Mean	43815	42692	44778	0.575
95% CI	40174–47455	37440–47,943	39712–49844	
Median	30899	28638	32997	
IQR	13508–48291	11576–45700	16499–49496	
**CPD, with stroke**				
Mean	75098	73743	76714	0.778
95% CI	64781–85415	60512–86973	60183–93246	
Median	53754	51630	57927	
IQR	22547–84962	16736–86524	32397–83457	
**COD, without stroke**				
Mean	36050	36725	35378	0.676
95% CI	32885–39215	31797–41654	31349–39408	
Median	27639	26629	30148	
IQR	13974–41305	12537–40722	13423–16726	
**Others**				
Mean	51571	51408	57315	0.993
95% CI	36167–66774	31086–71731	27016–76067	
Median	37403	37013	37792	
IQR	16759–58048	16210–57817	16183–59410	

* Inflation adjustments for all data pertaining to cost values across the 10-year study period were performed to 2015 US dollar values based on the Bureau of Labor Statistics Consumer Price Index (available at http://www.bls.gov/data/inflation_calculator.htm)

### Model derivation

Our model concluded several drivers of hospitalization cost (Table 3). Patients of Asian race undergoing bypass (20.1% higher compared to Caucasian), and those privately insured (21.6% higher compared to uninsured) were associated with increased hospitalization costs. Likewise, patients in the Northeast, Midwest, and West (29.8%, 33.1% and 65.1% higher as compared to south hospitals), and those at medium and high bypass case-load centers (24.1% and 51.0% more than low volume centers) were associated with higher costs. Adverse post-operative events following bypass surgery such as neurological complications including stroke (17.6% more), respiratory complications (21.6% more), ARF (56.4% more), or hyponatremia (25.3% more) were noted as potential drivers of costs. Likewise, hospital specific factors such as NDX and NPR were associated with increased hospitalization costs. A 1% increase in NDX or NPR resulted in 0.16% and 0.20% increase in hospital costs. Bypass procedural delay by a day following admission resulted in 3.2% increase in costs. Similarly, a day increase in LOS post bypass surgery caused 2.3% increase in hospital costs. On the contrary, elective procedure was noted to have 19.3% reduced hospital costs as compared to patients needing urgent bypass. With reference to patients with ruptured aneurysms and undergoing bypass surgery, those with moyamoya disease (25.8% lower), COD without infarction (32.2% lower), and with other indications including skull-base tumor encasing ICA (45.6% lower) were associated with lower costs. Our proposed model could explain the considerable proportion of variance in costs (R^2^ = 0.79). Based on our derived model, we propose a clinical app or calculator that could be utilized to predict the estimated cost of hospitalization for patients undergoing bypass (https://www.neurosurgerycost.com/calc/ec-ic-by-pass).

**Table 3:** Percent change in hospitalization cost following EC-IC bypass surgery for the variables included in the final predictive model.

Factors associated with increased cost	Percent change in cost	Factors associated with decreased cost	Percent change in cost
**Hospital-specific factors**		**Hospital-specific factors**	
NDX*	0.16	Elective admission	-19.3
NPR*	0.2	EC-IC indications**	
Post EC-IC bypass LOS†	2.3	MMD	-25.8
Days to EC-IC procedure†	3.2	COD without infarction	-32.2
**Patient demographics**		Other indication	-45.6
Asian race‡	20.1		
Private payer§	21.6		
Other payer§	34.2		
**Complications**			
Neurological complications including post bypass stroke	17.6		
Respiratory complications	21.6		
Hyponatremia	25.3		
ARF	56.4		
**Hospital characteristics**			
MVC∥	24.1		
HVC∥	51		
MW region#	29.8		
NE region#	33.1		
West region#	65.3		

*Numbers represent percent change in cost for 1% increase in the exposure variable;

^†^ Numbers represent percent change in cost for a day increase in the exposure variable;

In comparison with:

^‡^Caucasian race, and

^§^uninsured patients;

In comparison with hospitals with:

^∥^ low volume centers, and

^#^located in south region;

**In comparison with patients undergoing ECIC bypass for ruptured cerebral aneurysms

### Model validation

**Fig 2:**
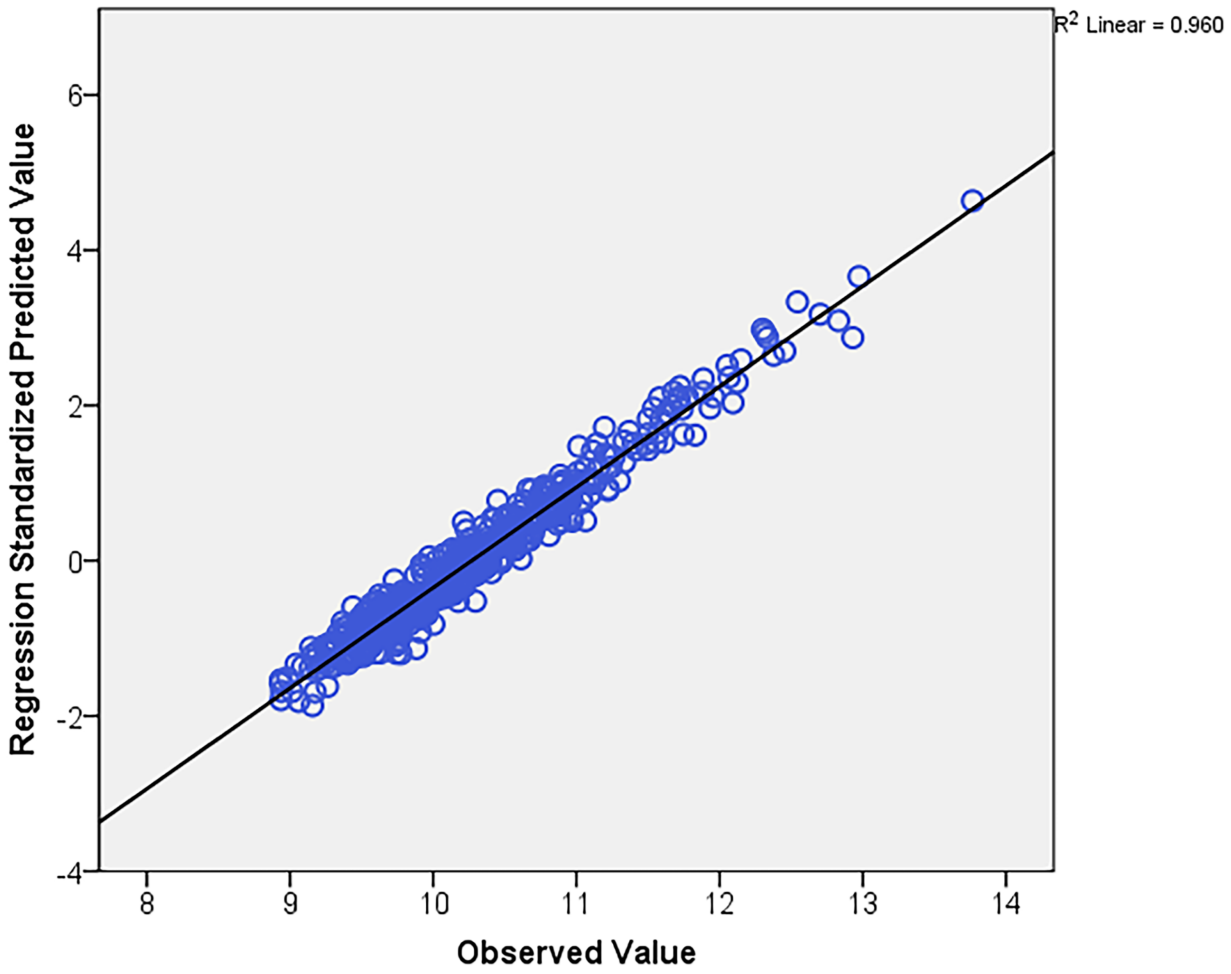
Scatterplot showing relationship of observed values with model predictive values of cost (ln transformed).

Our derived model was validated in a random, independent cohort of patients. A variation of less than 6% was noted in the derived R^2^ following model training (R^2^ = 0.75) from that of model testing. Our model demonstrated a significant strength of association (p<0.001) to predict in an independent cohort as assessed by testing model fit via plotting predicted values against observed values in the validation cohort (Fig 2).

## Discussion

The current study examines the impact of several patient and hospital characteristics on initial hospitalization cost for ECIC bypass surgeries. Modelled upon data obtained from the NIS over a 10-year span (2002–2011), we created a cost predictive app that serves to provide average estimation of hospitalization cost for bypass surgeries. In conjunction with patient safety and outcomes, cost-effectiveness is a critical aspect of healthcare delivery. Amidst the recent cost-containment debate, it is anticipated that procedures associated with high-cost and risks are likely to be the target of policy makers. The list of general hospital-acquired conditions (HACs), commonly known as “never events” compiled by the Centers for Medicare and Medicaid Services was expanded to include postoperative complications relating to certain surgical specialties. These include adverse thromboembolic events following joint replacement, surgical site infections (SSIs) following coronary artery bypass grafting, and SSIs following certain orthopaedic and bariatric procedures.[26, 27] Occurrence of these never events during hospital stay, thought to be averted via evidence-based medicine, imposes financial penalties on hospitals and providers that includes zero reimbursements for additional cost for managing these events, resulting in millions of dollars in loss each year. [27] These measures were partly to advocate higher patient safety levels and also to curtail hospitalization cost. Value-based incentives are likely to impact neurosurgical procedures, given the high-cost and risk associated with it. Cerebrovascular procedures such as aneurysm clipping, AVM resection, endovascular coiling, ECIC bypass, or carotid interventions are associated with inherent life threatening complications such as intraoperative rupture or graft failure that warrant acute attention, and intuitively impact total hospitalization cost. Wen et al estimated the occurrence of HACs for cerebrovascular procedures at 0.49%, with a relatively higher proportion for intracranial procedures compared to carotid interventions (1.33% vs 0.33%). Identifying modifiable targets likely to impact cost can direct preemptive measures towards mitigating it, and contribute to saving tax-payer dollars. Although several factors are logically known to impact cost, evidence-based quantification of estimates for neurosurgical procedures are limited. Previous studies proposed cost predictive models for patients undergoing aneurysm clipping or endovascular coiling for both ruptured and unruptured aneurysms.[28, 29] Utility of these models has also been explored in patients undergoing spinal procedure and brain tumor resection.[30, 31] These models serve as initial measures towards cost-containment in neurosurgery, albeit requiring future validation evaluating its validity using external datasets. Specific to bypass procedures, the focus of ongoing studies is dedicated towards assessing its safety and efficacy. To the best of our knowledge, no study performed a comprehensive analysis relating to hospitalization cost for bypass procedures. An indirect overview of the cost involved with bypass procedures in the treatment of unruptured giant cerebral aneurysms was reported by Familiari and colleagues based on data from 3 centers (2 in Germany, 1 in Italy). As a part of the Intracranial Aneurysm Study Group, the authors contrasted cost-effectiveness of endovascular versus surgical management of unruptured giant aneurysms, with bypass procedure constituting over 90% of the latter cohort.[32] The authors note that the direct treatment costs were significantly higher in the endovascular group as compared to the surgical cohort (median: $52,325 vs $20,619; p<0.01). [32] Although our analysis could not delineate between aneurysms based on their size (giant vs small and large), the median overall costs associated with unruptured aneurysms in the United States were dramatically higher compared to the estimates reported by Familiari et al (Median: $67,629 vs $20,619). This baseline comparison reiterates the high-costs associated with surgical management of cerebrovascular procedures, particularly bypass in the United States. Therefore, identification of factors driving hospitalization cost for these procedures are critical to measure the economic burden posed, and also to institute appropriate guidelines to curtail it. Further, assessments of these drivers can aid with in-hospital auditing, surgeon performance, and holds potential to redefine reimbursements.

Our study utilized robust methodological and statistical techniques for model development. Our model identified Asian race (20.1% higher) and those privately insured (21.6% higher) to be associated with higher cost of hospitalization as compared to Caucasian and those uninsured. Asians tend to have relatively higher rates of intracranial stenosis with concurrent atherosclerosis of intra and extracranial vessels.[33–37] Evidence suggests a genetic susceptibility, the presence of RNF213 gene on long-arm of chromosome 17, in Asian population for the high-incidence of moyamoya disease. [38–42] Estimates from a national study demonstrates Asians with moyamoya disease tend to have higher odds for increased LOS compared to Caucasian [OR: 1.37; 95% CI: 1.14–1.66; p<0.001], which has been cited as an obvious metric for cost increment. The association of private or commercial insurance with higher costs could be linked to the higher utilization of services in these patients. Bypass is a complex procedure that is associated with relatively high costs due to longer operative duration, hospitalization stay, repeated imaging, and monitoring. Compared to uninsured patients, the utilization of services is likely to be higher in privately insured patients. Hospitals located in the South region were associated with lowest hospitalization costs compared to Northeast, Midwest or West. Such geographical variation in cost has been noted in previous studies on cerebrovascular procedures.[28, 29] Although our observations cannot infer causality, in-depth analysis to identify drivers of costs across these regions and achieving levels comparable to lowest geographical region is warranted. Although hospital volume has a proxy for quality care in neurosurgery has often been debated[43], findings from most studies reflect high volumes to be associated with improved outcomes and reduced costs.[44–47] Interestingly, we found MVCs and HVCs to be associated with higher hospitalization cost as compared with LVC. This is plausibly linked to the complexity of the procedure that warrants technical precision and expertise, specialized centers with adequate infrastructure including well-equipped neuro-monitoring intensive care units, and a multidisciplinary team for management of intra or post-operative complications, and for catering long-term care. However, compared to LVCs, HVCs are associated with better outcomes in terms of mortality rates and several complications such as post-bypass neurological complications, acute renal failure, and thromboembolic events and tend to have shorter hospitalization stays for patients undergoing cerebral revascularization procedures. [48]

Hospital specific factors such as days to bypass, or post-procedural LOS, total NDX or NPR were also quantified to adversely impact hospital costs. LOS has been demonstrated to impact cost of cerebrovascular neurosurgical procedures previously.[28, 29] Prolonged LOS is intuitive of lack of adequate care, reflecting occurrence of adverse events necessitating additional care, and is likely to be targeted as a cost-containment measure. Further, we noted delay in performing bypass procedure can contribute to high costs, applicable in centers with limited surgeon volume and expertise to perform the procedure. Prompt cerebral revascularization is critical in patients with moyamoya angiopathy and selective cases of aneurysms. Interestingly, most patients with moyamoya are elective candidates for bypass. Predefined planning or elective admissions were thus noted to be associated with lower hospitalization costs. Emergent bypass indication, mostly intraoperatively, may uncontrollably increase costs. Serum electrolyte derangement, particularly lower sodium levels require repeated monitoring for correction, and are likely to impact costs. Further, we noted post-operative neurological complications including stroke, and respiratory complications to increase cost of care. These plausibly warrant further treatment, intensive monitoring and possibly invasive measures, and intuitively increase LOS. The development of renal failure as a predictor of costs is critical in this setting. Although indocyanine green angiography is now routinely being used, graft patency is often evaluated using iodine-contrast arteriograms or DSA. Sicker patients with multiple diagnoses on records, and those necessitating multiple procedures are likely to increase costs as quantified in our analysis. In contrast to ruptured aneurysms, patients with COD without stroke and moyamoya disease were associated to have lesser costs as they tend to require lesser functional, psychological and physical care. With the advent of endovascular techniques, the general consensus on performing cerebral revascularization on patients with ischemic stroke is often opposed, especially in the setting where randomized trials have failed to demonstrate superiority of bypass procedures over medical management. [4, 49] With approximately one-third patients undergoing cerebral revascularization for CODs, policy makers could potentially target reimbursements for bypass procedures to universally accepted indications (moyamoya disease, aneurysms etc) in the backdrop of cost-containment debate.

Despite obvious merits of our study, several limitations concerning the use of administrative databases apply to our investigation. For the years utilized, clinical comorbidities and complications in the data source were encoded using broad based ICD-9 coding definitions compared to the more granular ICD-10 definitions. Intrinsic information on the procedural technique known to impact outcomes such as the duration of surgery, intraoperative complications and blood loss, neuroimaging, and graft patency and hemodynamics were unavailable. Further, differentiation on the type of graft such as the low-flow (standard bypass or STA-MCA) versus the high-flow (eg. radial artery interposition graft) could not be differentiated. When indicated for aneurysms, the study was limited in terms of location and morphological metrics. For patients with SAH, data was limited in determining the severity or grading. Residual confounding and confounding by indication may account for some of the observed associations, and thus our model cannot gauge cost-variation in its entirety. However, it serves as a preliminary investigation in identifying baseline predictors of hospitalization cost for bypass. In the background of cost-containment reforms, our findings may be used as a baseline on which future studies could be formulated to identify potential low-cost targets. Based on our study design, our data cannot predict causality rather test associations. Lastly, coding inaccuracies including mis-coding or missed coding cannot be ruled out.

## Conclusions

Identified drivers of hospital cost after ECIC bypass could potentially be used as an adjunct for creation of data driven policies, impact reimbursement criteria, aid in-hospital auditing, and in the cost containment debate. Although, generalizability of our model can be inferred owing to the structure of the data source taking into account data from several non-federal hospitals spread over diverse geographical locations, clinical practice settings and payers, its applicability to US population at large needs further evaluation.

## Supporting information

S1 Table Coding definitions (ICD-9-CM codes). (PDF)

S2 Table Estimates of missing data for explanatory variables in the study cohort. (PDF)

S3 Table Association of explanatory variables included in the model development with ln(cost). (PDF)

S1 Fig Distribution of hospitals (n = 218) based upon number of EC-IC bypass procedures over study duration (10-yr period). (PDF)

S2 Fig Histogram showing distribution of regression standardized residuals in the (A) derivation and (B) validation cohort. (PDF)

S3 Fig P-P plot demonstrating the association of predicted versus observed residuals in the (A) derivation and (B) validation cohort. (PDF)

S4 Fig Scatter plot demonstrating relationship of regression standardized residuals against regression standardized predicted values for model derivation. (PDF)
