# Correction: Proposing a validated clinical app predicting hospitalization cost for extracranial-intracranial bypass surgery

**DOI:** 10.1371/journal.pone.0197200

**Published:** 2018-05-07

**Authors:** The PLOS ONE Staff

There is an error in affiliation 1 for authors Hai Sun, Piyush Kalakoti, Kanika Sharma, Jai Deep Thakur, Rimal H. Dossani, Devi Prasad Patra, Hesam Akbarian-Tefaghi, Frank Farokhi, Christina Notarianni, Bharat Guthikonda, and Anil Nanda. Affiliation 1 should be: Neurosurgery, Louisiana State University Health Sciences Center, Shreveport, Louisiana, United States of America. The publisher apologizes for this error.
