# Supplementary material for: Proposing a validated clinical app predicting hospitalization cost for extracranial-intracranial bypass surgery
Source: PLoS One. 2017 Oct 27;12(10):e0186758. doi: 10.1371/journal.pone.0186758 (PMC5659612; doi:10.1371/journal.pone.0186758)
Supplement: S1 Table — (PDF) [file pone.0186758.s001.pdf]

S1 Table. Coding Definitions (ICD-9-CM codes)

VARIABLES	CATEGORY	CODES AND DEFINITIONS	NOTES
**Procedure**	**Extracranial-intracranial (EC-IC) bypass surgery**	**39.28**	*ICD-9 Procedure code*
**Indications**	[a]**Moyamoya disease**	**437.5**	*ICD-9 Diagnosis code*
	[a]**Intracranial aneurysms:**<br>- ***Ruptured***<br>- ***Unruptured***	 **430**<br>**437.3**	*ICD-9 Diagnosis codes*
	[a]**Cerebro-occlusive disease:**<br>- ***Asymptomatic*** (without infarction)<br>- ***Symptomatic*** (with infarction)	 **433.00, 433.10, 433.20, 433.30, 433.80, 433.90, 434.10, 434.90, 437.0**<br>**433.01, 433.11, 433.21, 433.31, 433.81, 433.91, 434.01, 434.11, 434.91, 435, 435x, 437.1**	*ICD-9 Diagnosis codes*
	[a]**Others**	All others that underwent EC-IC bypass surgery except as mentioned above	-
**Independent (explanatory) variables**	**Model variables**		
**Patient demographics**	[b]Age	-	Metric
	[b]Gender	Male as reference	Qualitative
	[b]Race	Whites (ref), Blacks, Hispanics, Asians, other race	Qualitative
	[b]Payer	Self-payer (ref), Medicare, Medicaid, Private, Other payer	Qualitative
	[b]Median income quartiles	Lowest (ref), second, third, highest	Qualitative
	[b]Elective admission	Non-elective as reference	Qualitative
**Clinical characteristics**	[a]Indications for bypass	Ruptured (ref) and unruptured aneurysms, COD with and without stroke, moyamoya disease, and others	Qualitative (coding definitions above)

Patient comorbidities	TIA	**435, 435x**	*ICD-9 Diagnosis codes*
	Preop ischemic stroke	**430-432xx, 434.01, 434.11, 434.91**	*ICD-9 Diagnosis codes*
	Seizures	**345-345xx, 780.3-780.3x**	*ICD-9 Diagnosis codes*
	Anemia	**280-286x**	*ICD-9 Diagnosis codes*
	Coagulopathy	**287.1, 287.3-287.5, 287.8, 289.81, 289.82**	*ICD-9 Diagnosis codes*
	Hypercholesterolemia	**272-272.4**	*ICD-9 Diagnosis codes*
	Hypertension	**401-405xx**	*ICD-9 Diagnosis codes*
	CAD	**410-414xx**	*ICD-9 Diagnosis codes*
	COPD	**490-505x, 506.4**	*ICD-9 Diagnosis codes*
	CRF	**585-586250,**	*ICD-9 Diagnosis codes*
	DM	**250-250xx**	*ICD-9 Diagnosis codes*
	Alcohol abuse	**303.9, 303.9x, 305.0, 305.0x, V11.3**	*ICD-9 Diagnosis codes*
	Obesity	**278.00, 278.01**	*ICD-9 Diagnosis codes*
	Hyponatremia	**276.1**	*ICD-9 Diagnosis code*
Post-operative Complications	[a]Neurologic complications	**349.1, 349.3, 349.3x, 996.2, 996.75, 997.0, 997.0x**	*ICD-9 Diagnosis codes*
	[a]Respiratory complications	**518.5, 518.5x, 518.81, 518.82, 997.3, 997.3x**	*ICD-9 Diagnosis codes*
	[a]Cardiac complications	**410-411x, 997.1**	*ICD-9 Diagnosis codes*
	[a]Treated hydrocephalus	**02.2-02.3x**	*ICD-9 Procedure code*
	[a]Wound complications	**998.10, 998.1x, 998.3, 998.3x, 998.83**	*ICD-9 Diagnosis codes*
	[a]Acute renal failure	**584-584x**	*ICD-9 Diagnosis codes*
	[a]Pulmonary Embolism	**415.1, 415.1x, V12.51**	*ICD-9 Diagnosis codes*
	[a]Deep venous thrombosis	**451.1, 451.1x, 451.2, 451.81, 451.9, 453.2-453.4, 453.4x**	*ICD-9 Diagnosis codes*
		**453.6, 453.8, 453.8x, 453.9**	*ICD-9 Diagnosis codes*
		**38.7**	*ICD-9 Procedure code*
Hospital characteristics			
Hospital-level characteristics	[b]Hospital bedsize	Small (ref), medium, large	Qualitative
	[b]Hospital location/academic status	Rural (ref), urban non-teaching, urban teaching	Qualitative
	[b]Hospital region	South (ref), Northeast, Midwest, West	Qualitative
	[a]Hospital volume (weighted sample)	Low volume: ≤ 1/yr (ref), medium volume: 2-20/yr, high volume centers: ≥20/yr	Qualitative

Hospital specific factors	[b]Total number of diagnoses, NDX	-	Metric
	[b]Total number of procedures coded, NPR	-	Metric
	[a]Days to bypass	-	Metric
	[a]Post-procedural LOS	LOS minus Days to bypass	Metric
**Outcome measure (endpoints)**	[a]Total hospitalization cost	-	**Primary outcome**

[a]Data derived via computational analyses based on the data available from the HCUP files on NIS database [2002-2011];

[b]Based on the pre-existing data cataloged in the National Inpatient Sample database;
