# Supplementary material for: Proposing a validated clinical app predicting hospitalization cost for extracranial-intracranial bypass surgery
Source: PLoS One. 2017 Oct 27;12(10):e0186758. doi: 10.1371/journal.pone.0186758 (PMC5659612; doi:10.1371/journal.pone.0186758)
Supplement: S2 Table — (PDF) [file pone.0186758.s002.pdf]

S2 Table. Estimates of missing data for explanatory variables in the study cohort

Covariates	Proportion
Race	28.6%
Median income quartiles	2.8%
Gender	0.4%
Hospital bed-size	0.3%
Hospital academic status	0.3%
Age	0.1%
Elective admission	0.1%
Payer	0.1%
