# Supplementary material for: Proposing a validated clinical app predicting hospitalization cost for extracranial-intracranial bypass surgery
Source: PLoS One. 2017 Oct 27;12(10):e0186758. doi: 10.1371/journal.pone.0186758 (PMC5659612; doi:10.1371/journal.pone.0186758)
Supplement: S3 Table — (PDF) [file pone.0186758.s003.pdf]

S3 Table. Association of explanatory variables included in the model development with ln(cost)

	Explanatory variables	OR	95% Confidence interval		*P* value
**Patient demographics**	Age (metric)	1.00	1.00	1.00	0.708
	Female	1.02	0.94	1.10	0.619
	Whites	1.00	1.00	1.00	Reference
	African Americans	1.07	0.96	1.20	0.225
	Hispanics	0.99	0.85	1.15	0.880
	Asians	**1.22**	**1.06**	**1.41**	**0.007**
	Other race	0.93	0.76	1.13	0.444
	No insurance (self-payer)	1.00	1.00	1.00	Reference
	Medicare	1.19	0.95	1.50	0.130
	Medicaid	1.15	0.92	1.44	0.228
	Private insurance including HMO	**1.24**	**1.01**	**1.54**	**0.049**
	Other payers	**1.41**	**1.09**	**1.81**	**0.008**
	Lowest income quartile	1.00	1.00	1.00	Reference
	Second income quartile	1.03	0.92	1.15	0.585
	Third income quartile	1.06	0.95	1.18	0.304
	Highest income quartile	1.08	0.97	1.21	0.167
	Elective admission	**0.82**	**0.74**	**0.91**	**<0.001**
**Indication for ECIC bypass**	Ruptured aneurysms	1.00	1.00	1.00	Reference
	Unruptured aneurysms	0.95	0.73	1.23	0.701
	COD without stroke	**0.72**	**0.56**	**0.94**	**0.015**
	COD with stroke	0.80	0.62	1.04	0.093
	Moyamoya disease	**0.77**	**0.60**	**1.00**	**0.047**
	Other indications	**0.63**	**0.47**	**0.86**	**0.004**
**Patient comorbidities**	TIA	1.25	0.99	1.59	0.065
	Preop stroke	1.09	0.96	1.25	0.190
	Seizures	0.97	0.86	1.10	0.647
	Anemia	1.08	0.96	1.21	0.185
	Coagulopathy	1.02	0.80	1.31	0.856
	Hypercholesterolemia	0.96	0.87	1.05	0.351
	Hypertension	1.00	0.91	1.11	0.927

	CAD	1.00	0.88	1.12	0.935
	COPD	0.94	0.84	1.06	0.302
	CRF	0.85	0.62	1.16	0.295
	DM	1.07	0.97	1.18	0.167
	Alcohol Abuse	1.18	0.92	1.51	0.186
	Obesity	0.99	0.86	1.15	0.918
	Hyponatremia	**1.29**	**1.09**	**1.52**	**0.003**
**Postoperative complications**	Neurologic complications including stroke	**1.19**	**1.03**	**1.38**	**0.020**
	Respiratory complications	**1.24**	**1.05**	**1.47**	**0.012**
	Cardiac complications	1.03	0.83	1.28	0.771
	Hydrocephalus	1.06	0.85	1.32	0.606
	Wound complication	0.99	0.82	1.19	0.909
	ARF	**1.76**	**1.22**	**2.54**	**0.003**
	PE	0.96	0.71	1.28	0.761
	DVT	1.09	0.87	1.37	0.437
**Hospital characteristics**	Small bedsize	1.00	1.00	1.00	Reference
	Medium bedsize	0.98	0.80	1.22	0.883
	Large bedsize	1.13	0.95	1.35	0.176
	Rural	1.00	1.00	1.00	Reference
	Urban non-teaching	0.98	0.68	1.43	0.937
	Urban teaching	1.09	0.79	1.52	0.588
	South region	1.00	1.00	1.00	Reference
	Northeast region	**1.39**	**1.24**	**1.57**	**<0.001**
	Midwest region	**1.35**	**1.19**	**1.53**	**<0.001**
	West region	**1.92**	**1.72**	**2.14**	**<0.001**
	Low volume center	1.00	1.00	1.00	Reference
	Medium volume center	**1.27**	**1.08**	**1.50**	**0.005**
	High volume center	**1.67**	**1.37**	**2.02**	**<0.001**
**Hospital-specific factors**	*NDX (metric)	**1.17**	**1.08**	**1.27**	**<0.001**
	*NPR (metric)	**1.22**	**1.14**	**1.30**	**<0.001**
	Days to ECIC procedure(metric)	**1.03**	**1.02**	**1.04**	**<0.001**
	Post bypass LOS (metric)	**1.02**	**1.02**	**1.03**	**<0.001**
