# Supplementary material for: Proposing a validated clinical app predicting hospitalization cost for extracranial-intracranial bypass surgery
Source: PLoS One. 2017 Oct 27;12(10):e0186758. doi: 10.1371/journal.pone.0186758 (PMC5659612; doi:10.1371/journal.pone.0186758)

S1 Fig. Distribution of hospitals (n=218) based upon number of EC-IC bypass procedures over study duration (10-yr period)

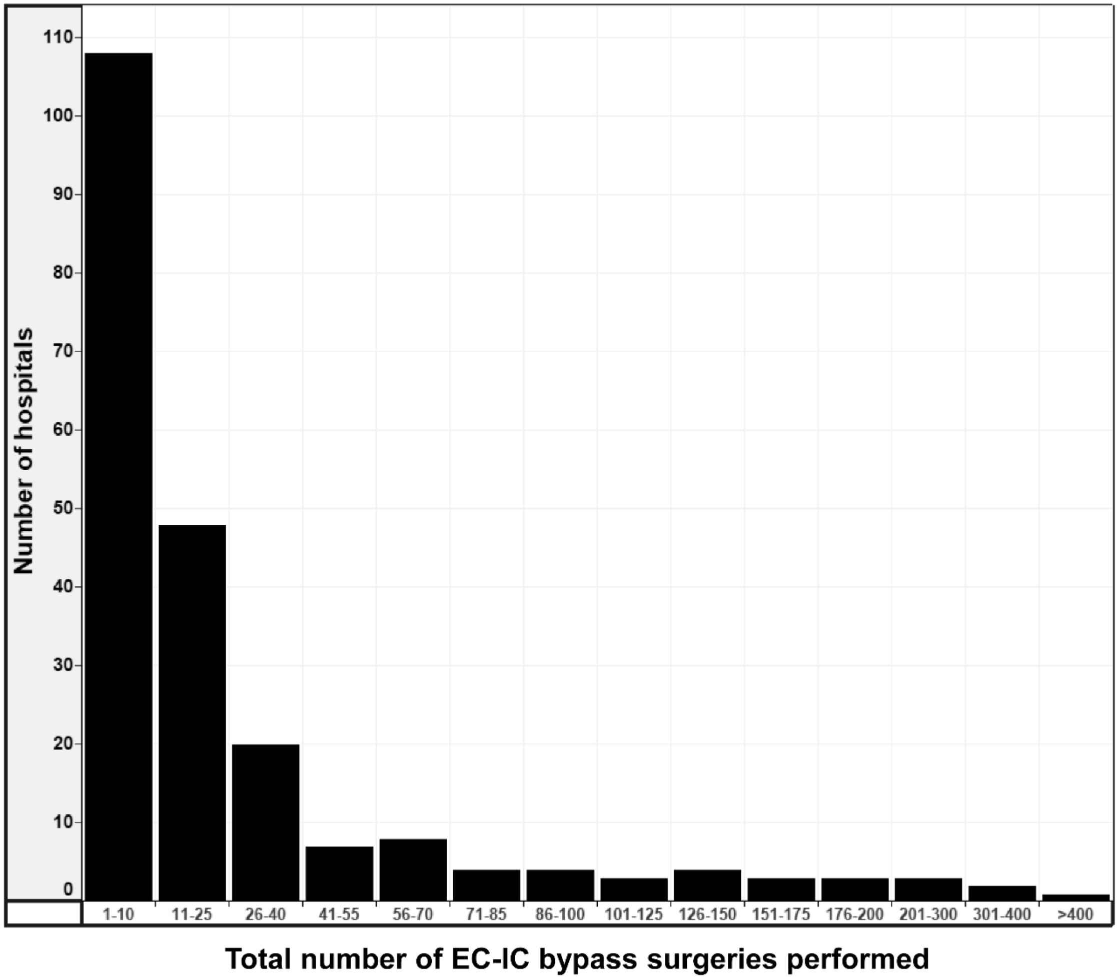

Supplement: S1 Fig — (PDF) [file pone.0186758.s004.pdf]
