# Supplementary material for: Proposing a validated clinical app predicting hospitalization cost for extracranial-intracranial bypass surgery
Source: PLoS One. 2017 Oct 27;12(10):e0186758. doi: 10.1371/journal.pone.0186758 (PMC5659612; doi:10.1371/journal.pone.0186758)
Supplement: S2 Fig — (PDF) [file pone.0186758.s005.pdf]

S2 Fig. Histogram showing distribution of regression standardized residuals in the (A) derivation and (B) validation cohort

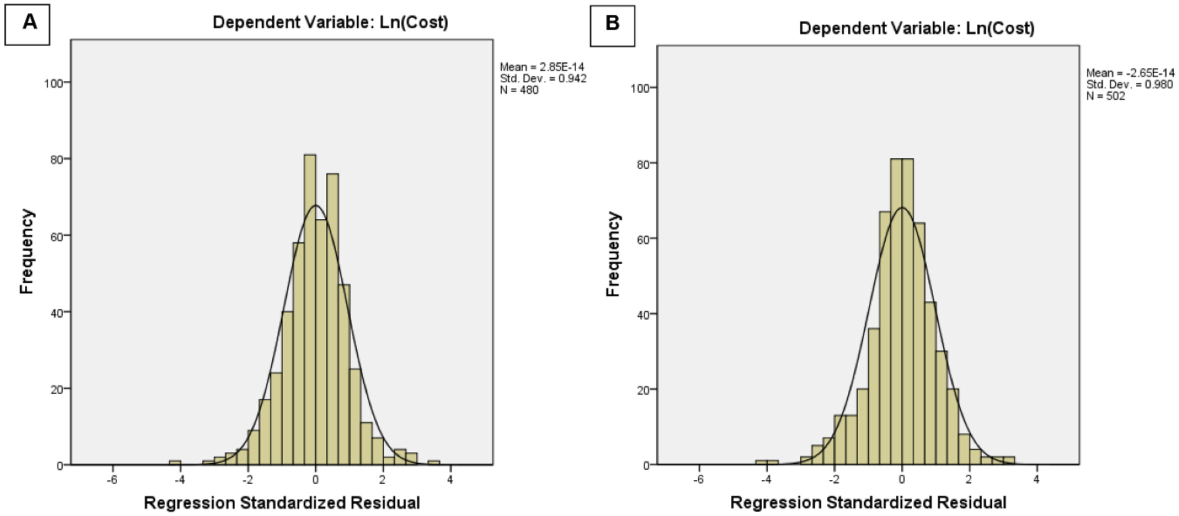

(Imputed dataset=5)
